# The impact of waiting time on patient outcomes: Evidence from early intervention in psychosis services in England

**DOI:** 10.1002/hec.3800

**Published:** 2018-07-16

**Authors:** Anika Reichert, Rowena Jacobs

**Affiliations:** ^1^ Centre for Health Economics University of York York UK

**Keywords:** mental health, psychosis, routine outcome measures, treatment intensity, waiting times

## Abstract

Recently, new emphasis was put on reducing waiting times in mental health services as there is an ongoing concern that longer waiting time for treatment leads to poorer health outcomes. However, little is known about delays within the mental health service system and its impact on patients. We explore the impact of waiting times on patient outcomes in the context of early intervention in psychosis (EIP) services in England from April 2012 to March 2015. We use the Mental Health Services Data Set and the routine outcome measure the Health of the Nation Outcome Scale. In a generalised linear regression model, we control for baseline outcomes, previous service use, and treatment intensity to account for possible endogeneity in waiting time. We find that longer waiting time is significantly associated with a deterioration in patient outcomes 12 months after acceptance for treatment for patients that are still in EIP care. Effects are strongest for waiting times longer than 3 months, and effect sizes are small to moderate. Patients with shorter treatment periods are not affected. The results suggest that policies should aim to reduce excessively long waits in order to improve outcomes for patients waiting for treatment for psychosis.

## 1 INTRODUCTION

Waiting times are a notable phenomenon in publicly funded health care systems such as the English National Health Service. Waiting lists can serve to stock available demand and optimise utilisation of the scarce supply of resources such as skilled staff and medical equipment (Culyer & Cullis, 1976). However, concerns arise when cases are affected in which waiting time may impede the patient's utility gain from the treatment. In the case of psychosis, timely access to care is considered a key priority in successful treatment. It has significant implications for the prevention of impairments and disabilities, functional, and symptomatic recovery, as well as the level of treatment engagement of patients (Doyle et al., 2014; Penttilä, Jääskeläinen, Hirvonen, Isohanni, & Miettunen, 2014). This is why recently, new emphasis has been placed on reducing waiting times in mental health services with the introduction of maximum waiting time targets for early intervention in psychosis (EIP) services in England (NHS England et al., 2015). But to date, little is known about delays within the mental health service system and their impact on patients.

This paper seeks to improve the understanding of the relationship between waiting times and patient outcomes in the context of EIP services in England. We investigate whether the time from acceptance onto the EIP caseload to start of treatment, not only leads to a deterioration in the patient's condition while waiting but also impedes the patient's ability to benefit from treatment up to 12 months after the start of treatment. The distinct feature of EIP services is that treatment is delivered over several months or years, and treatment intensity can vary from patient to patient. Further, recovery in psychosis is a long lasting process where keeping patients in a stable condition is considered a good outcome (Revier et al., 2015). Rather than looking at the outcomes immediately after a single treatment event as in previous literature, we look at patient outcomes after 12 months, incorporating treatment intensity during this time period. Our outcome measure, the Health of the Nation Outcome Scale (HoNOS) comes with a number of advantages for our analysis. Being clinician‐reported, it provides a measure of patient outcome, independent of the patient's subjectivity, which on the one hand is a desired dimension in patient‐reported measures (Fitzpatrick, Davey, Buxton, & Jones, 1998) but may be challenging for people with severe mental illness (McCabe, Saidi, & Priebe, 2007; Reininghaus & Priebe, 2012). Previous work on waiting times using other outcome measures consistently found low to moderate effect sizes—it is however questionable whether effects that are statistically significant, but small are also clinically relevant. We advance the analysis by estimating the impact of waiting time on a clinically reliable and significant change in HoNOS. Although HoNOS is not specific to psychosis, it is routinely collected in administrative data, which offers the potential to expand future analysis to other samples and mental health conditions in a comparable manner. As such, our work contributes to the literature discussing the feasibility and usefulness of routine outcome measures in general (Boswell, Kraus, Miller, & Lambert, 2015) and for mental health conditions in particular (Gilbody, House, & Sheldon, 2003; Tasma et al., 2017).

## 2 BACKGROUND

### 2.1 Theoretical framework

Waiting for treatment on a waiting list does not require patients to queue in person. Hence, there are no opportunity costs in terms of time spent waiting in order to clear markets. But still, waiting times impose costs, as introduced in the model of queuing by list by Lindsay and Feigenbaum (1984). Patients join the waiting list in order to obtain the right to receive treatment at some point in the future. The value of obtaining this right depends on the price to be paid when receiving the treatment (which is zero in the case of no copayments) and the delay (waiting time) to receive the good. The delay effects are composed of two factors: First, a positive interest rate will lead to a discounted value of the good consumed in the future relative to its present value. Second, the time of delivery affects the present value of consumption due to, for example, pain, uncertainty, and disability. In other words, the treatment received tomorrow is worth less because the patient (and caring relatives) have to experience suffering and inconvenience of living with a disease. Koopmanschap, Brouwer, Hakkaart‐van Roijen, and van Exel (2005) further demonstrated theoretically that the negative impact of waiting time can be long term. The deteriorated condition of the patient while waiting may take longer to recover or will not be reversed at all after a critical waiting time has passed. In the context of psychosis, the suffering can be significant. Psychoses summarise a group of serious mental health conditions in which a person's perception, thoughts, mood, and behaviour are significantly altered (NICE, 2014). Patients experience a high degree of impairment, and the often unusual or bizarre behaviour leads to difficulties in managing their own life up to the point of social exclusion (Huxley & Thornicroft, 2003). Although psychosis affects people in the most productive period of their working lives (mid‐1920s to late 1920s), they face lower rates of employment, lower payment, and less secure jobs (Marwaha & Johnson, 2004; Revier et al., 2015). All of the above suggests that waiting for psychotic treatment will contribute to a deterioration in treatment outcomes as symptoms and impairments will worsen over time and patients may disengage with treatment (Doyle et al., 2014; Penttilä et al., 2014).

### 2.2 EIP services and treatment

EIP services provide a full range of pharmacological, psychological, social, occupational, and educational interventions (NICE, 2014). The main treatment options are antipsychotic medication in conjunction with psychological interventions such as cognitive behavioural therapy and family interventions. The psychological interventions are delivered in a number of planned sessions over a period of at least 3 to 12 months. Given the multidisciplinary nature of EIP services, the care coordinator plays a key role in the effective delivery of EIP care (NHS England et al., 2015). They not only bring together all different professionals involved in the care of the patient, such as therapists, social workers, and psychiatrists. They are also responsible for engaging patients in treatment and supporting them across the total spectrum of their needs. Each EIP service is governed by the responsible mental health trust as the provider, which in turn negotiates the overall budget with a number of payers and distributes it to the individual services in charge.

### 2.3 Related literature

Two strands of literature shall be distinguished in the discussion of waiting times and outcomes. The first strand focuses on physical health conditions with most studies in the area of nonurgent surgical procedures such as hip and knee replacement (Braybrooke et al., 2007; Hamilton & Bramley‐Harker, 1999; Hamilton, Hamilton, & Mayo, 1996; Hirvonen et al., 2007; Hirvonen et al., 2009; Ho, Hamilton, & Roos, 2000; Nikolova, Harrison, & Sutton, 2016; Quintana et al., 2011; Tuominen et al., 2009, 2010), or more urgent surgical procedures such as organ transplantation (Meier‐Kriesche et al., 2000; Rauchfuss et al., 2013), and coronary artery bypass surgery (Manji, Jacobsohn, Grocott, & Menkis, 2013; Moscelli, Siciliani, & Tonei, 2016; Sari et al., 2007). Fewer studies investigate the relationship of waiting time with nonsurgical treatments such as rehabilitation (Collins, Suskin, Aggarwal, & Grace, 2015; Pedersen, Bogh, & Lauritsen, 2017), radiotherapy (Gupta, King, Korzeniowski, Wallace, & Mackillop, 2016; Noel et al., 2012; Seidlitz et al., 2015), or HIV treatment (Su et al., 2016). Results are inconsistent as to whether longer waiting causes worse chances of functional remission, recurrence, treatment adherence, quality of life, and mortality. Most of these studies use field data, which are limited in sample size, number of providers, and covariates to control for confounders. More recently, studies have used administrative data to overcome some of these limitations. Moscelli et al. (2016) found that waiting for coronary bypass surgery did increase the number of emergency readmissions but not in‐hospital mortality. Nikolova et al. (2016) analysed the impact of waiting for elective surgery on patient‐reported outcomes. They found that a longer waiting time reduces health‐related quality of life for hip and knee replacement but not for varicose veins and inguinal hernia.

The second strand of literature focuses on the impact of treatment delays on outcomes regarding first episode of psychosis patients. The key measure of waiting time in this context is the duration of untreated psychosis (DUP). DUP measures the time from the onset of the psychosis to the start of treatment and is mostly defined using patient interviews (Norman & Malla, 2001). Penttilä et al. (2014) recently published a comprehensive review of 33 studies. Longer DUP was associated with more severe symptomatic outcomes and reduced remission rates with small to moderate effect sizes. Also, longer DUP correlated with poorer social functioning but not with employment or quality of life. Some recent studies looked at long‐term effects of DUP on outcomes. In a 20‐year follow‐up, Cechnicki et al. (2014) found significantly deteriorated outcomes for the long DUP group (>6 months) in terms of symptom recovery, social functioning, and employment. Tang et al. (2014) reported significantly higher symptom remission rates for the shorter DUP group after accounting for confounding factors in a 13‐year follow‐up period. Despite the quantity of studies, evidence remains limited, because studies tend to be small‐scale with sample sizes between 23 and 776 patients using only a single or a few providers. Attrition rates ranged from 4% to 71% which could be a source of significant selection bias. Most studies are based on purely correlational methods or do not account adequately for the typically skewed nature of DUP (Marshall et al., 2005; Norman & Malla, 2001).

Our work aims to bridge the gap between these two distinct strands of literature. We advance the literature on psychotic patients by using well‐established methods from physical health care and a large, nationally representative sample. Our waiting time measure moves beyond the traditional concept of DUP to overcome some of its limitations. At the same time, we advance the literature in the physical health context by looking at a different treatment regime characterised by multiple treatment events over a period of several months. This stresses the importance of treatment intensity, which we include in the analysis.

## 3 DATA AND KEY MEASURES

**Figure 1:**
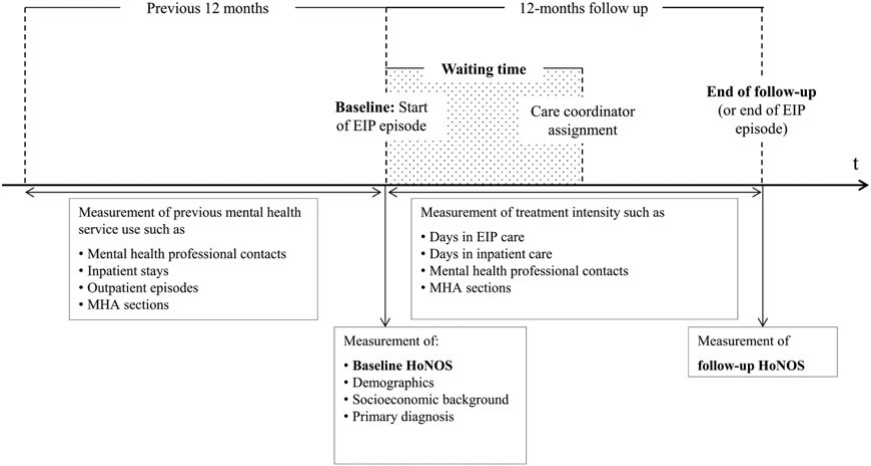
Study timeline with measurement points. EIP: early intervention in psychosis; HoNOS: Health of the Nation Outcome Scale

We use the Mental Health Services Data Set, which is a national administrative database of mental health‐related treatment in hospitals and community settings within the English National Health Service. Patients were included if they had a first EIP episode within the study period April 2012 to March 2014 and followed up for a period of 12 months. Figure 1 summarises the study timeline and measurement points.

Our outcome measure, HoNOS, is routinely collected by providers in our dataset (Wing et al., 1998; Wing, Curtis, & Beevor, 1999). HoNOS is composed of 12 items covering the four subdomains behaviour, impairment, symptoms, and social functioning (see Appendix A). Each item is evaluated by a trained clinician on a scale from 0 (*no problem*) to 4 (*severe to very severe problems*) and contributes equally to the total score ranging from 0 to 48. HoNOS measurements are conducted at treatment start and during the course of treatment. This allows us to observe a baseline HoNOS score at the start of the EIP episode and a score at follow‐up at the end of the 12 months (or at the end of the EIP episode if treatment ended before the follow‐up). We use the baseline measurement to condition on pretreatment severity. Further, we determine whether patients improved reliably and in a clinically meaningful way using the concept of reliable and clinically significant change introduced by Jacobson and Truax (1991) and applied to HoNOS by Parabiaghi, Barbato, D'Avanzo, Erlicher, and Lora (2005).

Inpatient waiting time as commonly used in physical health papers measures the time from the specialist's decision to treat until the start of the inpatient treatment (Siciliani, Moran, & Borowitz, 2014). We translate this concept to the context of psychosis by measuring the time from the patient's acceptance onto the EIP caseload (decision to treat) to the assignment of a care coordinator (start of treatment). The care coordinator is the key requirement for effective treatment to be initiated (NHS England et al., 2015). Previous papers found the relationship between waiting time and outcomes to be non‐linear with outcomes deteriorating significantly at a waiting time longer than 1 month (Tang et al., 2014) or 3 months (Cechnicki et al., 2014). Therefore, we employ three different transformations of waiting time: (a) a log transformation of waiting time in days, (b) waiting time quintiles with an equal number of patients in each group, and (c) waiting time intervals based on the thresholds typically used in the previous literature (0.5 to 3, 3 to 6, and 6 to 12 months).

## 4 METHODS

### 4.1 The model

We denote *h*_*ijkl*_ as the mental health status of the *i*th patient, *i* = 1, …, *N* who lives in small area *j*,  *j* = 1, …, *J* and receives treatment at provider *k*, *k* = 1, …, *K* in the financial year *l*, *l* = 1, …, *L*. The health status is measured prior to treatment ( $h_{\text{ijkl}}^{1}$) and 12 months after treatment start ( $h_{\text{ijkl}}^{2}$) as the total HoNOS score. Formally, the model is specified as follows:

(1) $$h_{\text{ijkl}}^{2}=\alpha W_{\text{ijkl}}+\beta h_{\text{ijkl}}^{1}+\gamma T_{\text{ijkl}}+\delta X_{\text{ijkl}}+\tau S_{\text{ijkl}}+y_{l}+u_{k}+\varepsilon_{\text{ijkl}},$$

where *W*_*ijkl*_ represents the patient's waiting time. The patient's outcome prospects are likely to depend on the severity of the condition at baseline. We therefore condition on the baseline HoNOS score $\;h_{\text{ijkl}}^{1}$. *T*_*ijkl*_ encompasses measures of treatment intensity. Over the 12‐month follow‐up period, treatment intensity will vary between patients but may also impact on the patient's outcomes. We approximate treatment intensity by the following variables: (a) the number of days in EIP care, (b) the number of days in inpatient care, and (c) the number of mental health professional contacts until the end of follow‐up (or end of EIP if earlier than follow‐up). We further control for whether a patient was being detained under the Mental Health Care Act in that period because additional legislative requirements impose a higher level of treatment intensity. As the degree to which each of the variables contributes to the patient's recovery process is unknown, we include each of them with equal weight into the model.

Patient characteristics that could impact both waiting time and outcomes are captured by *X*_*ijkl*_. Alongside a range of demographic characteristics, we consider the patient's socio‐economic background. At patient‐level, we include accommodation and employment status. Further, we used socio‐economic deprivation based on the Index of Multiple Deprivation measured at small‐area level (McLennan et al., 2011). Previous mental health service use, represented by *S*_*ijkl*_, may be indicative of the patient's ability to navigate through the system and take advantage of treatment options (and thus impact waiting times as well as outcomes). The vector includes the number of inpatient stays (in intervals 0, 1–2, >2), outpatient episodes (in intervals 0, 1–2, >2), mental health professional contacts (in intervals 0, 1–10, >10), and primary as well as secondary diagnoses within the 12 months prior to the EIP start. There are *L* unobservable year effects *y*_*l*_ and *K* unobservable provider‐level effects *u*_*k*_ for the 48 mental health trusts in our sample. The term *ε*_*ijkl*_ represents the idiosyncratic error.

Our main coefficient of interest is *α* that measures the effect of waiting time on follow‐up HoNOS outcomes conditional on the included covariates. We expect follow‐up outcomes to deteriorate if waiting time increases both because the waiting itself causes a worsening in the patient's condition and because the waiting impedes the patient's ability to benefit from treatment. Therefore, we expect a positive *α* indicating an increased (worse) follow‐up HoNOS score. By the application of provider and time fixed effects, any variation has to be interpreted as within provider variation for a given year.

Both the Shapiro–Wilk test (Shapiro & Wilk, 1965) and the Shapiro–Francia test (Shapiro & Francia, 1972) strongly rejected the null hypothesis of $h_{\text{ijkl}}^{2}$ being normally distributed. We accounted for the skewness by using generalised linear regression methods (Nelder & Wedderburn, 1972), which were shown to be an adequate choice in typically skewed data (Jones, Lomas, Moore, & Rice, 2016; Sinko, Turner, Nikolova, & Sutton, 2016). The modified Park test confirmed the Poisson distribution to fit the data best. Both the Pregibon link test (Pregibon, 1980) and the modified Hosmer–Lemeshow goodness‐of‐fit test (Hosmer & Lemeshow, 2005) accepted the square root link function. The Regression Equation Specification Error Test (Ramsey, 1969) further confirmed the model specification. We used cluster robust standard errors for the 48 mental health trusts (see Appendix C).

### 4.2 Robustness checks

We applied the same model from Equation 1 to each subdomain of HoNOS resulting in four separate models for behaviour, impairment, symptoms, and social outcomes. We estimated this system of linear equations as a seemingly unrelated regression model without constraints to account for cross‐model covariance, which was supported by the Breusch–Pagan Lagrange multiplier test for error independence (Zellner, 1962). Further, we use the concept of clinically significant and reliable change (see Appendix B for more details) to test whether the effect size we measure is of clinical relevance. We employed an ordered probit model to predict the impact of waiting time on the probability of a clinically significant and reliable change in the HoNOS score conditional on the same set of covariates as introduced above.

## 5 RESULTS

### 5.1 Descriptive statistics

We identified 14,912 patients (full sample) having a first EIP episode and a care coordinator within the study period. We excluded 5,874 patients (39.4%) for which we could not observe two complete HoNOS records. Another 89 patients (0.01%) were excluded, which were from providers treating fewer than 30 patients in the sample. The remaining study sample included 8,949 patients being treated within 48 mental health trusts. Table 1 compares key characteristics of the study sample with those from the full and the excluded sample.

**Table 1:** Descriptive statistics

	Full sample	Study sample	Excluded sample
Number of patients	14,912	8,949	5,963
Number of providers	55	48	55
Patient demographics			
Patient age (mean)	24.9	25.8	23.6
Male (%)	63.7	63.6	64.0
White ethnicity (%)	73.4	72.1	75.6
Marital status: Single (%)	88.5	87.1	90.9
Schizophrenia diagnosis (%)	53.6	52.9	55.2
Socio‐economic background			
Mainstream housing (%)	83.5	83.6	83.3
Unemployed (%)	45.2	47.4	41.1
Least deprived quintile (%)	9.9	9.3	10.8
Most deprived quintile (%)	37.8	38.1	37.3
Mental health service use (before start of EIP care)			
Zero health professional contacts (%)	31.7	18.3	52.0
Zero outpatient episodes (%)	73.7	68.0	82.3
Zero inpatient admissions (%)	69.2	60.2	82.8
Zero Mental Health Care Act section (%)	77.5	71.6	86.5
HoNOS score at baseline (mean)			
Total (min 0, max 48)	14.0	14.1	13.5
Behaviour score (min 0, max 12)	2.8	2.8	2.9
Impairment score (min 0, max 8)	1.4	1.2	1.0
Symptoms score (min 0, max 12)	5.7	5.7	5.5
Social score (min 0, max 16)	4.4	4.4	4.1
Treatment intensity (during EIP care)			
Days in EIP care (mean)	291.5	306.6	268.9
Days in inpatient care (mean)	15.2	18.8	9.9
Mental health professional contacts (mean)	36.6	42.5	27.7
Mental Health Care Act sectioned (%)	20.5	23.9	15.3

*Note*. HoNOS observations are reported for the total study sample and for *n* = 10,012 in the full sample and *n* = 1,063 in the excluded sample. EIP: early intervention in psychosis; HoNOS: Health of the Nation Outcome Scale.

**Figure 2:**
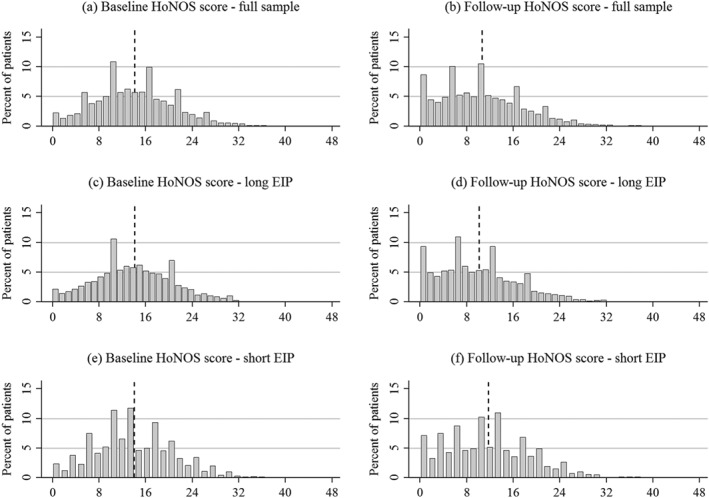
Histogram of HoNOS scores at baseline and follow‐up. EIP: early intervention in psychosis; HoNOS: Health of the Nation Outcome Scale

Our study sample was on average 25.8 years old, predominantly male, of White ethnicity, single, and diagnosed with schizophrenia. Most lived in mainstream housing within the most deprived neighbourhoods and were unemployed. The mean HoNOS score at baseline was 14.1. Figure 2 visualises the distributional shift of HoNOS scores towards zero from baseline to follow‐up. During the 12 months follow‐up, patients in the study sample spent on average 18.8 days in inpatient care and experienced 42.5 contacts with any kind of mental health professional; 23.9% were sectioned under the Mental Health Care Act at least once during the time of follow‐up. Our study sample was on average 2 years older than the excluded patients and more likely unemployed. Most evident is that patients in the study sample were more likely to have been in contact with mental health services in the previous 12 months. Also, treatment intensity during the EIP care was higher for the study sample. Mean HoNOS scores at baseline were, however, very similar on all dimensions.

We note that not all patients spent the total follow‐up time in EIP care. We therefore stratified the study sample by whether a patient finished EIP care before the end of follow‐up (“short EIP” group, 31.4%) or not (“long EIP” group, 68.6%) and run analyses for the two subsamples separately. Table 2 shows that in all three samples, HoNOS decreased (improved) from baseline to follow‐up by about two to four points. The short EIP group improved less in HoNOS but waited almost 15 days longer than the long EIP group.

**Table 2:** Summary statistics of waiting time and HoNOS

	Study sample		Long EIP		Short EIP	
	Mean	*SD*	Mean	*SD*	Mean	*SD*
Baseline HoNOS	14.1	6.8	14.1	6.8	14.0	6.8
Follow‐up HoNOS	10.6	7.0	10.1	6.9	11.7	7.2
Waiting time	50.1	74.1	42.1	64.1	67.4	89.8
Observations	8,949		6,135		2,814	

*Note*. EIP: early intervention in psychosis; HoNOS: Health of the Nation Outcome Scale.

**Figure 3:**
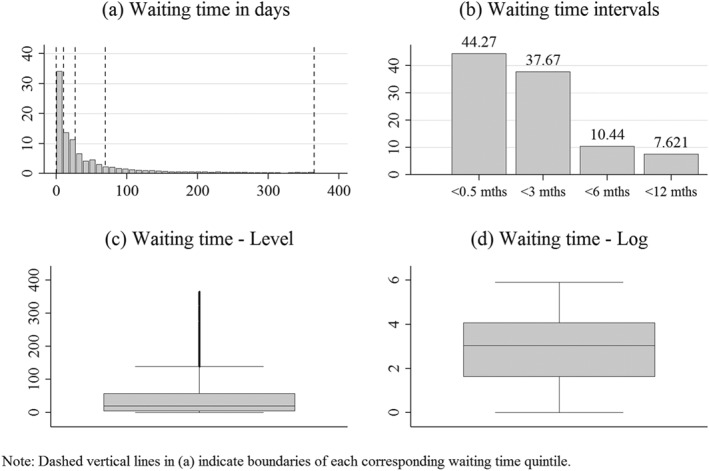
Descriptive statistics on waiting time

Figure 3 summarises several descriptive statistics of our main explanatory variable, waiting time. As expected, we find waiting time to be heavily right skewed with a median of 20 and a mean of 50 days. Consequently, the largest proportion of patients was allocated to the waiting time interval of less than 0.5 months (panel [b]). We also see that taking the logarithm of waiting time helps to reduce a large amount of the skewness (panels [c] and [d]).

**Figure 4:**
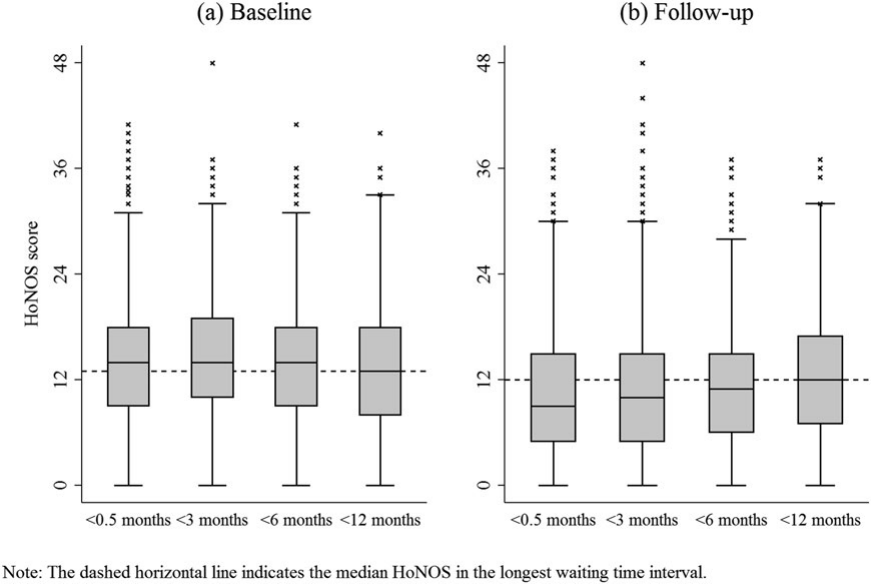
Box plots for HoNOS scores by waiting time intervals

Figure 4 visualises the distribution of HoNOS scores across the different waiting time intervals. Baseline HoNOS scores in panel (a) vary very little across intervals of waiting particularly for the first three intervals. Although patients from the longest waiting interval had the lowest median HoNOS at baseline, they improved least at follow‐up. Panel (b) shows that median follow‐up scores decreased (improved) most at follow‐up for the shorter waiting time intervals.

### 5.2 Estimation results

Table 3 displays the estimation results from the regression of Equation 1 including marginal effects (dy/dx). The estimates for the three different waiting time measures result from three independent regressions. Model (1) includes the whole study sample whereas Models (2) and (3) look at long and short EIP patients, respectively. We observe a significant but small effect of log waiting time on the HoNOS score 12 months after the EIP start for the total sample and the long EIP group. A 1% longer waiting time translates into an increase (worsening) in HoNOS by 0.20 to 0.27 points. The association between longer waiting and worse outcomes is only significant for the longest waiting quintile—however with a larger effect than the overall. Being in the longest waiting quintile is associated with a 0.78 to 1.27 points higher (worse) HoNOS compared to the shortest waiting time quintile. For long EIP patients, we observe a clear gradient looking at the waiting time intervals. Patients waiting between 0.5 and 3 months (3 to 6 months; 6 to 12 months) had a 0.34 (1.15; 1.61) higher HoNOS score than patients waiting less than 0.5 months. Patients with an EIP episode shorter than the follow‐up time seem to be not significantly affected by the length of waiting.

**Table 3:** Generalised linear model results with follow‐up HoNOS as dependent variable

	(1) Study sample			(2) Long EIP			(3) Short EIP		
	Coeff.	*SE*	dy/dx	Coeff.	*SE*	dy/dx	Coeff.	*SE*	dy/dx
Log waiting time (continuous)	0.032***	(0.009)	0.20	0.043***	(0.010)	0.27	0.022	(0.015)	0.15
Waiting time quintiles (ref.cat.: shortest quintile)									
2nd shortest quintile	−0.050	(0.036)	−0.32	−0.015	(0.049)	−0.09	−0.109	(0.064)	−0.73
3rd shortest quintile	−0.057	(0.041)	−0.36	0.004	(0.051)	0.02	−0.186**	(0.060)	−1.24
4th shortest quintile	0.031	(0.033)	0.20	0.050	(0.039)	0.31	0.036	(0.081)	0.25
Longest quintile	0.119**	(0.045)	0.78	0.199***	(0.053)	1.27	0.021	(0.072)	0.14
Waiting time intervals (ref.cat.: less than 0.5 month)									
Waiting time 0.5 to 3 months	0.040	(0.023)	0.25	0.054*	(0.027)	0.34	0.034	(0.045)	0.23
Waiting time 3 to 6 months	0.120***	(0.034)	0.78	0.181***	(0.048)	1.15	0.076	(0.052)	0.52
Waiting time 6 to 12 months	0.215***	(0.059)	1.41	0.250***	(0.065)	1.61	0.189	(0.098)	1.30
Observations	8,949			6,135			2,814		
Provider and year fixed effects	yes			yes			yes		
Covariates	yes			yes			yes		

*Note*. Model (1) includes the complete study sample. Model (2) includes only patients with an EIP episode longer than the follow‐up. Model (3) includes only patients with an EIP episode shorter than the follow‐up. “dy/dx” represents average marginal effects in days. For factor levels, they present the discrete change from reference category. All models use cluster robust standard errors for 48 provider clusters. EIP: early intervention in psychosis.

* *p* < 0.05.

** *p* < 0.01.

*** *p* < 0.001.

Table 4 reports the estimated coefficients and marginal changes of the baseline HoNOS as well as the treatment intensity variables based on Equation 1. As expected, we observe a strong positive relationship between baseline and follow‐up HoNOS scores. A higher or worse baseline condition strongly predicts worse outcomes 12 months after treatment start. Most severely affected patients had an up to five points worse outcome at follow‐up. Overall, treatment intensity does not seem to impact outcomes much. Although significant, effect sizes are small. More days spent in EIP care seem to improve outcome prospects. Interestingly, more days of inpatient care and more mental health professional contacts are associated with a deterioration in follow‐up outcomes. This may be explained by the fact that the two variables also capture some level of baseline severity of the patient that is not captured in the other control variables. In this case, more severe patients would need more inpatient care and service contacts but at the same time have worse outcome prospects regardless of treatment intensity. For the short EIP group, we observe patients who were sectioned under the Mental Health Care Act to have significantly worse outcomes. This again may be explained by the variable capturing some different dimension of baseline severity, but it may also be an indication that involuntary treatment worsens outcome prospects.

**Table 4:** Generalised linear model results of baseline HoNOS and treatment intensity on follow‐up HoNOS

	(1) Study sample		(2) Long EIP		(3) Short EIP	
	Coeff.	dy/dx	Coeff.	dy/dx	Coeff.	dy/dx
Baseline HoNOS (ref.cat.: least severe)						
2nd least severe quintile	0.220***	1.34	0.154***	0.92	0.347***	2.18
3rd least severe quintile	0.310***	1.92	0.268***	1.63	0.404***	2.57
4th least severe quintile	0.400***	2.51	0.297***	1.82	0.620***	4.08
Most severe quintile	0.544***	3.49	0.449***	2.82	0.759***	5.09
Treatment intensity						
Number of days in EIP care	−0.001***	−0.01	—	—	—	—
Number of days in inpatient care	0.001*	0.00	0.001**	0.01	0.000	0.00
Number of mental health professional contacts	0.005***	0.03	0.005***	0.03	0.004***	0.03
Mental Health Care Act sectioned within follow‐up	0.023	0.15	−0.023	−0.15	0.153**	1.04
Observations	8,949		6,135		2,814	
Provider and year fixed effects	yes		yes		yes	
Covariates	yes		yes		yes	

*Note*. All models include log waiting time as regressor. EIP: early intervention in psychosis; HoNOS: Health of the Nation Outcome Scale.

* *p* < 0.05.

** *p* < 0.01.

*** *p* < 0.001.

### 5.3 Robustness of results

Results from the effects of waiting time on the different HoNOS subdimensions are provided in Appendix D. In line with previous findings, we find the strongest negative impact of waiting time on the symptoms dimension. But also, all other subdimensions are negatively affected by a longer waiting time. As before, patients waiting longer than 3 months are affected most by a deterioration in outcomes on each subdomain. Although effect sizes are moderate, we find evidence of a significant increase in the probability of a reliable and clinically significant deterioration for the study sample and the long EIP group. The likelihood of a clinically relevant deterioration is again highest for the longest waiting patients (see Appendix E).

## 6 DISCUSSION

Waiting times for mental health services in general and for EIP services in particular have recently gained considerable policy interest. But little is known about the detrimental effect of delays within the care system on outcomes for patients with psychosis. We document a moderate decline in patient outcomes 12 months after treatment acceptance for additional days of waiting. Despite moderate effect sizes, the risk of a clinically significant and reliable deterioration is elevated by longer waiting time. Effects are significant in the waiting time range from 3 to 12 months, which supports the threshold theory discussed in previous papers. Also consistent with previous literature, all outcome dimensions are affected with the largest impact on symptomatic and social outcomes.

Our study contributes a number of aspects to existing evidence. First, we developed a strategy to measure a system‐related waiting time measure in contrast to the commonly used DUP. DUP has been criticised in its suitability to measure service effectiveness as definitions vary considerably across studies and are prone to a self‐report bias by patients (Norman & Malla, 2001; Register‐Brown & Hong, 2014; Singh, 2007). Our waiting time measure allows us to investigate the impact of delays within the care system rather than the help‐seeking behaviour of patients (Gronholm, Thornicroft, Laurens, & Evans‐Lacko, 2017). Second, we consider treatment intensity during the time of follow‐up. It allows us to reflect recovery in psychosis as a long lasting process and patient outcomes as a result of repeated service contacts over a period of several months. Finally, we are the first to study a routine outcome measure (HoNOS) to look at psychosis outcomes. HoNOS has been found to have adequate or good validity, reliability, sensitivity to change, and feasibility (Amin et al., 1999; McClelland, Trimble, Fox, Bell, & Stevenson, 2000; Pirkis et al., 2005; Wing et al., 1998). Given its generic nature, it may lack clinical precision. But our findings are consistent with studies that use specific but heterogeneous outcome measures.

We note some limitations of our work. First, we may have underestimated waiting time as we excluded any waiting time that occurred between the first service contact (e.g., general practitioner) or self‐referral and the specialist's decision to treat. If longer waiting time does indeed have negative effects on outcomes, we would have estimated a lower bound of the effect. Second, we restricted our follow‐up period and thus treatment intensity to 12 months given the boundaries of data availability. Longer follow‐up has, however, been shown to increase the impact of waiting time on outcomes (Penttilä et al., 2014). If this is the case, then again, our results are a lower bound estimation. Third, our outcome measure demonstrates the clinician's judgement of the patient's condition, which may not necessarily match the patient's perception (Kramer, Owen, Wilson, & Thrush, 2003). Fourth, this work is limited by the relatively high number of missing HoNOS records, which is common when working with clinician‐reported measures (Jacobs, 2009). The remaining study sample had substantially higher proportions of mental health service contacts prior to the EIP treatment than excluded patients. This would have limited the external validity of our results if the relationship between waiting for treatment and outcomes of the same treatment would be different dependent on past service experience. On the one hand, patients may have learned coping strategies during previous service contacts, which help them to deteriorate less during the time of waiting. On the other hand, patients with more service contacts in the past may be in a more severe condition overall, which will worsen even more during waiting. Whereas in the first case, we would have underestimated the negative impact of waiting time, we would have overestimated it if the latter case is true. Without further knowledge about the role of previous service use in the interplay of waiting time and outcomes, our results have to be interpreted as representative for a patient cohort with relatively high mental health service use in the past. If there were systematic differences in HoNOS coding quality between providers, which in turn may be associated with the provider's performance regarding patient waiting times and outcomes, we have controlled for these through the use of provider fixed effects.

Finally, the estimated effect is based on the assumption that the baseline health outcome conditional on other individual characteristics, including previous service use and treatment intensity, is sufficient to account for the individual's unobserved pretreatment severity. We find the baseline outcome to be a strong predictor for the follow‐up outcome. Also, accounting for previous service use and treatment intensity may have captured some remaining severity not observed by the baseline HoNOS. However, there may still have remained unobserved severity that explains both longer waiting times and worse outcomes. Future research should aim to consider either a valid instrument or a suitable comparison group to deal with this challenge.

Our results have direct implications for the recently introduced waiting time target policy for EIP services. As has been the case in many previous target policies in other health areas, the 14‐day target appears to have been chosen arbitrarily rather than based on evidence. A comprehensive discussion on the optimal targeted waiting time needs to consider the effects on patient outcomes but also implications for the supply side. Our paper sheds some light on the demand dimension. According to our results, the target policy can only be effective in improving patient outcomes if it leads to a reduction in excessive waits longer than 3 months.

## CONFLICT OF INTEREST

None.

## FUNDING

Anika Reichert conducted this research as part of her PhD study that is funded by a scholarship from the Centre for Health Economics, University of York.

## APPENDIX A HEALTH OF THE NATION OUTCOME SCALE (HONOS) ITEMS, SUBDOMAINS, AND TOTAL SCORE

**Table:**

HoNOS item	HoNOS subscore	Total HoNOS score
1. Aggression	A: Behaviour (max. score = 12)	Total HoNOS score (max. score = 48)
2. Self‐harm		
3. Drug and alcohol use		
4. Cognitive problems	B: Impairment (max. score = 8)	
5. Physical illness and disability		
6. Hallucinations and delusions	C: Symptoms (max. score = 12)	
7. Depression		
8. Other symptoms		
9. Relationships	D: Social (max. score = 16)	
10. Activities in daily living		
11. Residential environment		
12. Day‐time activities		

*Note*. Based on Wing et al. (1998) and Wing et al. (1999).

## APPENDIX B RELIABLE AND CLINICALLY SIGNIFICANT CHANGE

For more details, we refer the interested reader to Jacobson and Truax (1991) and Parabiaghi et al. (2005). A reliable and clinically significant change satisfies two criteria: (1) a clinically significant change would move a person from a score typical of the “dysfunctional” population to a score typical of the “functional” population, and (2) a reliable change is beyond what could be attributed to measurement error or chance.

1. Clinically significant change

Patients were defined as dysfunctional if they had a score of ≥3 in at least two of the 12 items. All others made up the functional population. The cut‐off point where there is equal chance of belonging to either population (dysfunctional or functional) is calculated as follows:

$$\mathrm{cut}-\mathrm{off}\;=\frac{\left({\text{mean}}_{\text{dysfunc}}\times {\mathrm{SD}}_{\text{func}}\right)+\left({\text{mean}}_{\text{func}}\times {\mathrm{SD}}_{\text{dysfunc}}\right)\;}{\left({\mathrm{SD}}_{\text{func}}\times {\mathrm{SD}}_{\text{dysfunc}}\right)}=\frac{\left(17.9\times 4.5\right)+\left(8.9\times 5.5\right)\;}{\left(4.5+5.5\right)}=13.0.$$

A change of at least 13 score points in HoNOS was considered as clinically significant.

1. Reliable change

We calculated a reliable change index (*RC*_index_) based on the baseline HoNOS score:

$${\mathrm{RC}}_{\text{index}}=1.96\times {\mathrm{SE}}_{\text{diff}}=1.96\times 5.2=10.2,$$

where *SE*_diff_ is the standard error of measurement of a difference

$${\mathrm{SE}}_{\text{diff}}={\mathrm{SD}}_{1}\times \sqrt{2}\times \sqrt{1-\alpha }=6.8\times \sqrt{2}\times \sqrt{1-0.71}=5.2.$$

*SD*_1_ is the standard deviation of the baseline score and α is Cronbach's coefficient. A change of at least 10 score points in HoNOS was considered as reliable.

Based on this, we classified patients with a given HoNOS at baseline (*score*_1_) and follow‐up (score_2_) as improved if *score*_2_ ≥ *RC*_improv_, where *RC*_improv_ = *score*_1_ + *RC*_index_ and deteriorated if *score*_2_ ≤ *RC*_deter_, where *RC*_deter_ = *score*_1_ − *RC*_index_. In the study sample, 77.4% were classified as stable, 18.7% improved, and 3.9% deteriorated.

## APPENDIX C GENERALISED LINEAR MODEL DIAGNOSTICS—DEPENDENT VARIABLE: TOTAL HONOS SCORE AT FOLLOW‐UP

**Table:**

Test for normality of follow‐up HoNOS					
	Obs	*W*	*V*	*z*	Prob > *z*
Shapiro–Wilk test	8,949	0.98	103.33	12.38	0.000
Shapiro–Francia test	8,949	0.98	109.33	12.32	0.000

Within/between provider variance in follow‐up HoNOS					
	Mean	*SD*	Min	Max	Obs
Overall variance	50.01	74.1	0	365	*N* = 8,949
Between provider variance		29.1	0	133.0	*n* = 48
Within provider variance		69.5	−82.9	380.7	T‐bar = 186.44

GLM model diagnostics	based on model with independent variable: log waiting time				
Log pseudolikelihood	−36006				
Squared correlation btw. y and yhat	0.183				
Linktest yhat	*p* > |t| = 0.000				
Linktest yhat squared	*p* > |t| = 0.051				
Hosmer–Lemeshow test	*F*(10, 8939) = 0.42; Prob > *F* = 0.9385				
Ramsey RESET test	χ^2^(1) = 5.78; Prob > χ^2^ = 0.0162				
Park test	Gaussian		χ^2^(1) = 26.12; Prob > χ^2^ = 0.0000		
	Poisson		χ^2^(1) = 2.20; Prob > χ^2^ = 0.1376		
	Gamma		χ^2^(1) = 4.59; Prob > χ^2^ = 0.0322		
	Inverse Gaussian		χ^2^(1) = 33.26; Prob > χ^2^ = 0.0000		

## APPENDIX D SEEMINGLY UNRELATED REGRESSION RESULTS—DEPENDENT VARIABLE: HONOS SUBSCORE AT FOLLOW‐UP

**Table:**

	(1) Behaviour		(2) Impairment		(3) Symptoms		(4) Social	
	Coeff.	*SE*	Coeff.	*SE*	Coeff.	*SE*	Coeff.	*SE*
Log waiting time (continuous)	0.040**	(0.014)	0.025**	(0.009)	0.078***	(0.018)	0.061**	(0.021)
Waiting time quintiles (ref.cat.: shortest quintile)								
2nd shortest quintile	−0.084	(0.076)	−0.084	(0.049)	−0.044	(0.100)	−0.157	(0.117)
3rd shortest quintile	−0.111	(0.076)	−0.009	(0.048)	−0.060	(0.099)	−0.218	(0.117)
4th shortest quintile	0.055	(0.077)	0.076	(0.049)	0.068	(0.100)	0.004	(0.118)
Longest quintile	0.145	(0.077)	0.053	(0.049)	0.341***	(0.101)	0.198	(0.118)
Waiting time intervals (ref.cat.: less than 0.5 month)								
Waiting time 0.5 to 3 months	0.066	(0.048)	0.089**	(0.031)	0.074	(0.063)	0.045	(0.074)
Waiting time 3 to 6 months	0.146*	(0.074)	0.078	(0.047)	0.295**	(0.096)	0.259*	(0.114)
Waiting time 6 to 12 months	0.314***	(0.085)	0.154**	(0.054)	0.516***	(0.111)	0.419**	(0.130)
Observations	8,949		8,949		8,949		8,949	
Provider and year fixed effects	yes		yes		yes		yes	
Covariates	yes		yes		yes		yes	

*Note*. The dependent variable is in Model (1) the HoNOS behaviour subscore (Items 1–3), in Model (2) the HoNOS impairment subscore (Items 4–5), in Model (3) the HoNOS symptoms subscore (Items 6–8), and in Model (4) the HoNOS social subscore (Items 9–12).

* *p* < 0.05.

** *p* < 0.01.

*** *p* < 0.001.

## APPENDIX E ORDERED PROBIT ESTIMATION RESULTS—DEPENDENT VARIABLE: RELIABLE AND CLINICALLY SIGNIFICANT CHANGE IN TOTAL HONOS SCORE

**Table:**

	(1) Study sample		(2) Long EIP		(3) Short EIP	
	Coeff.	*SE*	Coeff.	*SE*	Coeff.	*SE*
Log waiting time (continuous)	0.033**	(0.012)	0.042**	(0.014)	0.029	(0.020)
Waiting time quintiles (ref.cat.: shortest quintile)						
2nd shortest quintile	−0.066	(0.061)	−0.052	(0.080)	−0.083	(0.109)
3rd shortest quintile	−0.117*	(0.058)	−0.042	(0.075)	−0.312**	(0.108)
4th shortest quintile	−0.012	(0.054)	−0.020	(0.063)	0.080	(0.127)
Longest quintile	0.137*	(0.064)	0.220**	(0.083)	0.042	(0.101)
Waiting time intervals (ref.cat.: less than 0.5 month)						
Waiting time 0.5 to 3 months	−0.023	(0.040)	−0.024	(0.046)	0.017	(0.069)
Waiting time 3 to 6 months	0.122	(0.063)	0.216*	(0.084)	0.048	(0.091)
Waiting time 6 to 12 months	0.253***	(0.077)	0.290**	(0.096)	0.239*	(0.116)
Observations	8,949		6,135		2,814	
Provider and year fixed effects	yes		yes		yes	
Covariates	yes		yes		yes	

*Note*. Models (1)–(3) are ordered probit models with the dependent variable defined as follows: 1 = clinical improvement, 2 = clinical stability, and 3 = clinical deterioration. Model (1) includes the complete study sample. Model (2) includes only patients that were in EIP care for the whole duration of follow‐up. Model (3) includes only patients with an EIP episode shorter than the 12‐month follow‐up. All models use cluster robust standard errors for 48 provider clusters.

* *p* < 0.05.

** *p* < 0.01.

*** *p* < 0.001.
